# Plasma Levels of Matrix Metalloprotease MMP-9 and Tissue Inhibitor TIMP-1 in Caucasian Patients with Polypoidal Choroidal Vasculopathy

**DOI:** 10.3390/vision4020027

**Published:** 2020-05-15

**Authors:** Jakob Ø. Sørensen, Yousif Subhi, Christopher R. Molbech, Marie Krogh Nielsen, Torben L. Sørensen

**Affiliations:** 1 Clinical Eye Research Division, Department of Ophthalmology, Zealand University Hospital, Vestermarksvej 23, DK-4000 Roskilde, Denmark; c.molbech@gmail.com (C.R.M.); mariekroghnielsen@gmail.com (M.K.N.); tlso@regionsjaelland.dk (T.L.S.); 2 Department of Ophthalmology, Rigshospitalet-Glostrup, DK-2600 Glostrup, Denmark; 3 Faculty of Health and Medical Science, University of Copenhagen, DK-2200 Copenhagen, Denmark

**Keywords:** polypoidal choroidal vasculopathy, plasma, biomarker, matrix metalloproteinase

## Abstract

Background: Matrix metalloproteinase 9 (MMP-9) and tissue inhibitor of metalloproteinases 1 (TIMP-1) are regulating enzymes of the extracellular matrix. A systemic imbalance of MMP-9 and TIMP-1, thought to reflect an imbalance of the extracellular matrix homeostasis, is previously associated with polypoidal choroidal vasculopathy (PCV) in Asian patients. Previous studies suggest inter-ethnical differences in the genetic background and etiology of PCV. To further explore this issue, we studied the plasma levels of MMP-9 and TIMP-1 in Caucasian patients with PCV and compared to healthy age-matched controls. Methods: For this prospective case-control study, 60 participants were recruited who were either patients with PCV (*n* = 26) or healthy controls (*n* = 34). All participants underwent detailed clinical examination. We sampled fresh venous blood, isolated plasma, and quantified plasma concentrations of the extracellular matrix regulators MMP-9 and TIMP-1 using electrochemiluminescence immunoassays. Results: Plasma levels of MMP-9 (*p* = 0.4), TIMP-1 (*p* = 0.9), and MMP-9/TIMP-1 ratio (*p* = 0.4) did not differ significantly between patients with PCV and healthy controls. No differences appeared after adjusting for influencing co-variates in multivariate analyses. Conclusion: We demonstrate that Caucasian patients with PCV do not have altered levels of plasma MMP-9 or plasma TIMP-1. These findings suggest no strong evidence of a systemic imbalance of the extracellular matrix homeostasis in Caucasian patients with PCV. Our findings are in line with studies of other aspects of PCV that are also subject to significant inter-ethnical differences.

## 1. Introduction

Polypoidal choroidal vasculopathy (PCV) is a distinct clinical entity characterized by polypoidal abnormalities of vessels originating from the choroid, also described as aneurismal type 1 neovascularization in the literature [1,2]. It is an important differential diagnosis to neovascular age-related macular degeneration (AMD) since the accepted standard treatment of neovascular AMD is monotherapy using intravitreal injections of vascular endothelial growth factor inhibitors (anti-VEGF), whereas the accepted standard treatment of PCV is based on a combination therapy using and full-dose photodynamic therapy to achieve polyp closure and anti-VEGF [1,3,4]. In some cases, monotherapy with either photodynamic therapy or anti-VEGF may suffice for PCV. Although several clinical features may suggest a presence of PCV—such as slightly younger age of presentation, presence of orange-red subretinal nodules, dome-shaped retinal pigment epithelium (RPE) detachments on optical coherence tomography (OCT) scans, or hemorrhagic presentation—none of these features decisively differentiates PCV from neovascular AMD [1,3,5]. Gold standard for diagnosis of PCV remains indocyanine green angiography (ICGA), which can reveal polyps as well as any associated branching vascular networks [1,3,5]. 

An important characteristic of PCV is its strong inter-ethnical differences in clinical presentation and genetic background [5,6]. In Asians, PCV is present in approximately 1 out of 2 patients with suspected neovascular AMD undergoing ICGA for diagnostic examination, and a higher proportion of patients with PCV are male [6]. In comparison, PCV is present in 5%–10% of Caucasians with suspected neovascular AMD, and no strong gender association is observed [5]. One important difference in clinical presentation is that PCV is found in the macula in the majority of Asian patients [7], whereas the majority of Caucasian patients present with lesions located peripherally or peripapillary [5]. These inter-ethnical differences in location also explain why Asian patients present with a worse initial best-corrected visual acuity (BCVA) but also experience a stronger treatment response on BCVA, whereas Caucasian patients with the extrafoveal and peripapillary lesions present with better initial BCVA but experience a more attenuated treatment response on BCVA [8,9,10]. Genetically, one interesting inter-ethnical difference is that PCV associates with a single nucleotide polymorphism (rs2301995) in the elastin gene in Japanese [11,12], but not in Caucasians [13]. Immunologically, Zeng et al. reported one of the strongest biomarkers associated with PCV: serum matrix metalloproteinase 9 (MMP-9) was twice as high in Chinese patients with PCV compared to healthy controls (equivalent of a Cohen’s *d* of 1.0, indicating a very large difference) [14]. MMP-9 is a zinc-dependent endopeptidase in humans with proteolytic effect on extracellular matrix proteins such as elastin and collagen. Zeng et al. also measured tissue inhibitor of metalloproteinases 1 (TIMP-1, also known as metalloproteinase inhibitor 1), which is a glycoprotein that functions as the specific inhibitor of MMP9. TIMP-1 did not differ between the groups [14]. Hence, in Asian patients with PCV, a higher MMP-9 and no difference in TIMP-1 points towards an imbalance in the extracellular matrix homeostasis. Considering the inter-ethnical differences in the elastin gene and association to PCV [11,12,13], which may be suggestive of an etiological difference in the involvement of extracellular matrix, we hypothesized if the association between PCV and MMP-9 would be present in Caucasian patients with PCV. Hence, our aim with this prospective clinic-based case-control study was to quantify plasma levels of MMP-9 and its inhibitor tissue inhibitor of metalloproteinases 1 (TIMP-1, also known as metalloproteinase inhibitor 1) in patients with PCV and compare to healthy age-matched control individuals in a Caucasian population.

## 2. Materials and Methods

### 2.1. Study Design

We followed the Declaration of Helsinki, local ethical approval was obtained (jr. no. SJ-379), the nature of the study was explained a priori, and we obtained informed consent from all participants. Power calculations was based on the Zeng et al. study [14], and assuming α = 0.05 and β = 0.80, which identified that a sample size of 16 in each group would suffice. We recruited at least 20 participants in each group for a total of 60 participants.

### 2.2. Participants

We consecutively recruited patients with PCV and age-matched healthy control individuals with healthy maculae. Patients with PCV had at least one eye with at least one hyperfluorescent polyp with a hypofluorescent halo seen in early-phase ICGA with or without associated branching vascular networks. Other features of PCV were included to support a diagnosis but were not compulsory: orange-red focal subretinal polyp-like structures on fundus photographs and pulsating polyps on ICGA-video [15]. Healthy controls had <10 small drusen (diameter <63 μm), did not present with any signs of pigment abnormalities, and were recruited from biologically unrelated healthy relatives of the patients to better match groups in possible environmental exposures. To avoid systemic influence of immunological co-morbidities, we did not include participants with any history of active cancer or any autoimmune, infectious, or inflammatory diseases. We measured plasma C-reactive protein to ensure no acute systemic immune activation, which was defined as >15 mg/L [16].

### 2.3. Participant Recruitment and Data Collection

All participants were subject to an interview. We noted medical history and lifestyle habits. All medical data were validated and cross-checked using the electronic patient record. Lifestyle habits included alcohol consumption (weekly consumption estimated as units per week, where units are defined as 1 unit = 12 mL ethanol), being regularly physically active [17], and smoking (current, previous (>100 cigarettes during lifetime and ceased for >12 months), or never). Body mass index was calculated using height and weight. We sampled venous blood from antecubital veins for immediate use. Blood was collected in lithium heparin coated tubes and centrifuged for 15 min at 1500 G to separate plasma, and then plasma was isolated and stored at −80 °C for later quantification in batches. 

### 2.4. Quantification of Plasma MMP-9 and TIMP-1

Plasma MMP-9 and TIMP-1 were quantified using the commercially available U-PLEX Human assays from MSD (U-PLEX Human, Meso Scale Diagnostics, Rockville, MD, USA), which is an electrochemiluminescent-based multiplex immunoassay. We prepared assays as per the manufacturer’s instructors and recommendations. Plasma samples were run in duplicate and diluted 10-fold in the MMP-9 assay and 50-fold in the TIMP-1 assay. Prepared plates were read using the QuickPlex SQ120 (Meso Scale Diagnostics, Rockville, MD, USA). Because of our duplicate measurements, we were able to calculate the coefficient of variation for each sample and re-analyzed those with a too high coefficient of variation defined as more than 20%. Values for analysis were averaged values from the duplicate measurements.

### 2.5. Data Analysis and Statistics

We analyzed data in SPSS v.23.0.0.0 (IBM Corp., Armonk, NY, USA). Continuous variables were presented and compared using methods according to whether or not normal distribution was present. Since plasma MMP-9, plasma TIMP-1, and the MMP-9/TIMP-1 ratio were not normally distributed, we used the Mann–Whitney U-test for comparisons between groups. Categorical variables were compared using χ^2^ test or the Fisher’s exact test for small categories (defined as <5 in each cell). Univariate and multivariate ordinal logistic regression analyses were made to explore associations beyond retinal diagnosis and to adjust for any significantly influencing factor.

## 3. Results

We recruited 60 participants for this study. Twenty-six were patients with PCV and 34 were healthy control individuals (Table 1). All participants were white Caucasian Danes. Fewer patients with PCV were never smokers (*p* = 0.03). Apart from smoking habits, groups did not differ significantly.

**Figure 1:**
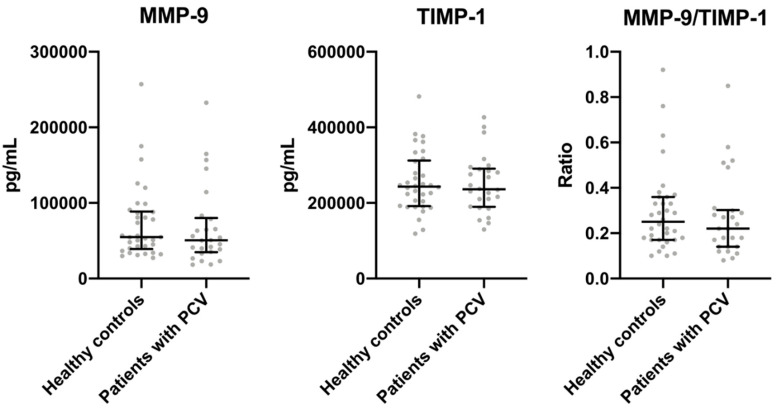
Plasma levels of matrix metalloproteinase-9 (MMP-9), tissue inhibitor of metalloproteinase-1 (TIMP-1), and MMP-9/TIMP-1 ratio in Caucasian patients with polypoidal choroidal vasculopathy (PCV) and healthy controls. Comparisons were made using the Mann–Whitney U-test. Groups did not differ significantly (*p* > 0.05 for all comparisons).

Patients with PCV and healthy control did not differ in plasma MMP-9 (*p* = 0.4), TIMP-1 (*p* = 0.9), or MMP-9/TIMP-1 ratio (*p* = 0.4) (Figure 1).

Female gender was associated with lower levels of plasma MMP-9 (OR 0.36; CI 95% 0.15–0.90; *p* = 0.03). Adjusting for female gender did not change the conclusion that MMP-9 did not differ between patients with PCV and healthy controls (*p* = 0.4). Increasing age was associated with higher levels of plasma TIMP-1 (OR 1.15; CI 95% 1.08–1.23; *p* < 0.001). Adjusting for age did not change the conclusion that TIMP-1 did not differ between patients with PCV and healthy controls (*p* = 0.6). We found no association between co-variates investigated and MMP-9/TIMP-1 ratio, hence no meaningful adjustment could be done. Taken together, we found no association between PCV and altered plasma levels of MMP-9, TIMP-1, or the MMP-9/TIMP-1 ratio (Table 2). 

## 4. Discussion

Results of this study are the first reported plasma levels of MMP-9 and TIMP-1 in Caucasian patients with PCV. We find no significant differences between PCV and healthy controls. Our findings add to the increasing pool of evidence suggesting that PCV is subject to a great amount of inter-ethnical differences in its etiology [3,5,6,7,8,9,10,11,12,13]. 

Our study design does not allow conclusions on whether or not MMP-9 vs. TIMP-1 imbalances play a role in local chorioretinal disease development—but only that a general imbalance in extracellular matrix homeostasis is not present in Caucasians with PCV. It is important to distinguish systemic levels from local expression and activity in the choroid. In a histopathological study of human PCV specimens, MMP-9 was highly present in the choroidal vessel walls of the polypoidal lesions [18]. A major source of MMP-9 is immune cells and their presence around PCV lesions have been reported in several histopathological studies [18,19,20,21,22,23]. In an experimental study, Kumar et al. demonstrated that expression of proteases in RPE lead to degradation of elastin in the choroid mimicking features of PCV, and further progression required complement activation and immune cell infiltration [18]. Taken together, these findings are suggestive of a local degradation and matrix remodeling, and our findings in this study suggest that such activity, at least in Caucasian patients, are not present on a systemic level. The reason to why immune cells infiltrates and secretes MMP-9 remain incompletely understood, but it appears that changes or potential dysfunction in the circulating immune cells may be one contributing factor of disease etiology [24,25,26,27,28,29]. However, at this point, very few etiological studies are published and further studies are warranted to better understand the etiological mechanisms of PCV.

Strengths and limitations of this study should be noted. Zeng et al. measured MMP-9 and TIMP-1 in serum [14], whereas we measured in plasma. This introduces a difference between the studies which makes direct comparisons difficult. Although serum and plasma levels of these proteins do correlate [30], plasma levels are more accurate and experts discourage the use of serum for measurement since the extent of coagulation and fibrinolysis is directly related to the measured MMP-9 activity in serum [31,32,33]. Another strength of this study is our strategy to match the control group to our study group in environmental exposures, which otherwise can influence measures of systemic immunology including MMP-9 [34,35]. We also addressed this issue by including multivariate analyses. In these analyses, gender significantly influenced MMP-9 levels. We were unable to address this issue a priori, e.g., by gender-matching our control group, since this was not known to us prior to the study. It is also important to note that we only measured MMP-9 and TIMP-1, and our results cannot be used to infer on the activity of other proteins and enzymes involved in extracellular matrix homeostasis. It should be noted that a limitation to this study is that we did not perform retinal angiography on healthy controls. Finally, a limitation to our cross-sectional study design is that we can only find associations, whereas establishment of causal relationships requires an experimental study design.

In conclusion, we here demonstrate that Caucasian patients with PCV do not have alterations in plasma MMP-9 or plasma TIMP-1. Although these proteins involved in the extracellular matrix homeostasis may play a role locally in the choroid, we find no strong evidence of a systemic imbalance.

## Figures and Tables

**Table 1:** Participant characteristics.

Characteristics	Patients with PCV (*n* = 26)	Healthy Controls (*n* = 34)	*p*-Value
Age, years, mean (SD)	72 (7)	74 (8)	0.4 ^†^
Gender, males:females, *n*	11:15	15:19	0.9 ^‡^
Cardiovascular disease, *n* (%)	5 (19)	5 (15)	0.6 ^‡^
Hypertension, *n* (%)	10 (39)	11 (32)	0.6 ^‡^
Hypercholesterolemia, *n* (%)	9 (35)	12 (35)	1.0 ^‡^
Type 2 diabetes mellitus, *n* (%)	3 (12)	0 (0)	0.08 ^§^
Alcohol consumption, units, median (IQR)	4 (1 to 13)	4 (2 to 7)	0.8 ^¶^
Body mass index, mean (SD)	25 (4)	25 (3)	1.0 ^†^
Physically active, *n* (%)	14 (54)	22 (65)	0.4 ^‡^
Smoking, current:previous:never, *n*	8:15:3	5:15:14	0.03 ^§^

Abbreviations: PCV = polypoidal choroidal vasculopathy; SD = standard deviation; IQR = interquartile range. Symbols: ^†^ Independent samples t-test. ^‡^ χ^2^ test. ^§^ Fisher’s exact test. ^¶^ Mann–Whitney U-test.

**Table 2:** Univariate and multivariate analyses to explore possible associations between polypoidal choroidal vasculopathy (PCV) and the plasma levels of matrix metalloproteinase-9 (MMP-9) and tissue inhibitor of metalloproteinase-1 (TIMP-1) as well as the MMP-9/TIMP-1 ratio.

	**MMP-9**				**TIMP-1**				**MMP-9/TIMP-1 Ratio**	
	***Univariate*** ***OR; CI95%***	***p-Value***	***Multivariate*** ***OR; CI95%***	***p-Value***	***Univariate*** ***OR; CI95%***	***p-Value***	***Multivariate*** ***OR; CI95%***	***p-Value***	***Univariate*** ***OR; CI95%***	***p-Value***
Retinal diagnosis										
PCV	0.65; 0.27–1.59	0.3	0.66; 0.27–1.61	0.4	0.93; 0.38–2.25	0.9	1.28; 0.52–3.11	0.6	0.67; 0.28–1.63	0.4
Healthy	*Ref.*		*Ref.*		*Ref.*		*Ref.*		*Ref.*	
**Participant characteristics included as co-variates**										
Age	0.95; 0.99–1.12	0.09			1.15; 1.08–1.23	<0.001	1.16; 1.08–1.24	<0.001	0.99; 0.94–1.05	0.7
Gender										
Female	0.36; 0.15–0.90	0.03	0.36; 0.14–0.90	0.03	0.48; 0.20–1.19	0.1			0.61; 0.25–1.49	0.3
Male	*Ref.*		*Ref.*		*Ref.*				*Ref.*	
Alcohol	1.00; 0.94–1.06	0.9			0.97; 0.92–1.03	0.4			0.99; 0.93–1.05	0.8
BMI	1.02; 0.90–1.14	0.8			1.08; 0.96–1.22	0.2			0.97; 0.86–2.41	0.6
PA	1.03; 0.42–2.52	0.9			0.79; 0.32–1.93	0.6			1.01; 0.41–2.46	1.0
Smoking										
Current	1.84; 0.52–6.48	0.3			0.84; 0.24–2.92	0.8			1.94; 0.55–6.86	0.3
Previous	1.65; 0.59–4.66	0.3			1.95; 0.69–5.52	0.2			0.99; 0.35–2.79	1.0
Never	*Ref*.				*Ref.*				*Ref.*	
CVD	1.14; 0.35–3.69	0.8			0.55; 0.17–1.79	0.3			1.52; 0.47–4.94	0.5
HT	0.81; 0.32–2.02	0.6			1.59; 0.63–4.02	0.3			0.56; 0.22–1.42	0.2
HC	0.76; 0.30–1.92	0.6			0.49; 0.19–1.26	0.1			1.15; 0.46–2.88	0.8
T2DM	0.94; 0.13–7.02	1.0			3.13; 0.41–23.8	0.3			0.46; 0.06–3.47	0.5

When any co-variates were significantly associated in the univariate analyses, they were included in the multivariate analyses. This was the case when looking at plasma levels of MMP-9 and TIMP-1, but not for the MMP-9/TIMP-1 ratio; hence, a multivariate analysis for the latter would show exact same statistics as the univariate and therefore not presented. Presented statistics for the co-variates are indicative of per age increase in year, having female gender, per weekly unit increase in alcohol consumption, per unit increase in body mass index (BMI), being physically active (PA), being previously or currently smoker, or having any of the prevalent lifestyle co-morbidities: cardiovascular disease (CVD), hypertension (HT), hypercholesterolemia (HC), or type 2 diabetes mellitus (T2D). Abbreviations: BMI = body mass index; CI95% = 95% confidence interval; CVD = cardiovascular disease; HT = hypertension; HC = hypercholesterolemia; T2DM = type 2 diabetes mellitus; MMP-9 = matrix metalloproteinase 9; OR = odds ratio; PA = physically active; PCV = polypoidal choroidal vasculopathy; TIMP-1 = tissue inhibitor of metalloproteinase.
